# The organ-specific modulation of malignant processes by opioids: a systematic review of cell culture studies

**DOI:** 10.1038/s41416-025-03014-4

**Published:** 2025-04-11

**Authors:** Natalia Hefteh, Olivia Welch, Mahdi Sheikh, Adam La Caze, Marie-Odile Parat

**Affiliations:** 1 School of Pharmacy, The University of Queensland, St Lucia, QLD Australia; 2 Genomic Epidemiology Branch, International Agency for Research on Cancer, Lyon, France

**Keywords:** Cancer models, Cell growth

## Abstract

**Background:**

The International Agency for Research on Cancer (IARC) monographs recently classified opium consumption as *carcinogenic to humans* in certain organs, raising concerns regarding the effects of pharmaceutical opioids. This systematic review (Open Science Framework osf.io/xyg9p) evaluated whether opioid exposure causes organ-specific modulation of malignant processes in cancer cell culture studies.

**Methods:**

We identified all research articles evaluating tumour modulation by opioids in vitro through 28/02/2024. Data on the organ of origin of cancer cells, opioid activity, opioid exposure, and cancer outcomes (tumour cell growth, metastasis, clonogenicity) were extracted. Statistical analyses were performed using Fisher’s exact tests and multivariable logistic regression.

**Results:**

The anti-cancer outcome was the most prevalent (57%) while only 11% of experiments reported pro-cancer outcomes. A total of 230 publications, comprising 1465 experiments, were included. Low opioid concentrations (*p* = 0.0005), short exposure durations (*p* = 0.0035), and organs where cancer risk increases with opium use (*p* = 0.002), were associated with reporting of pro-cancer effects for opioid agonists.

**Conclusion:**

The findings support a positive association between opioid exposure and growth of cancer cells from lung, bladder, larynx, pancreas, pharynx, stomach, or oesophagus and further reveal duration and concentration as critical factors in experiments evaluating the effects of opioids on cultured cancer cells.

## Introduction

Opioids are widely used in the management of cancer-induced pain as well as during the perioperative period of cancer surgery [1]. Despite their widespread use, the role of opioids in tumorigenesis, tumour growth, and metastasis remains controversial [2]. Through interactions with their associated receptors on tumour cells, opioids exhibit various effects on cellular processes critical to tumorigenesis [1].

The literature contains evidence in support of both pro- and anti-tumour effects of opioids [2], with multiple mechanisms proposed. Opioids have been shown to promote cancer progression through immunosuppression *via* the reduction of natural killer (NK) cytotoxicity [3, 4] and T cell activity [5], induction of pro-inflammatory responses mediated by mu opioid receptor (MOR) activation [6], stimulation of angiogenesis by increasing endothelial cell (EC) proliferation and survival [7], enhanced tumour growth *via* pro-proliferative and anti-apoptotic mechanisms [7], and promotion of cancer cell migration and invasion *via* reduction of cell adhesion and regulation of chemotaxis [8]. Contrasting evidence suggests that opioids have anti-tumour properties. Opioids alleviate pain, thereby mitigating the tumour-promoting stress response associated with cancer or cancer surgery [9]. They exhibit anti-inflammatory effects through kappa opioid receptor (KOR) activation [6] and inhibit angiogenesis by inducing endothelial cell apoptosis [10], decreasing endothelial cell motility, and reducing hypoxia-mediated effects [11]. Opioids further impede tumour growth through anti-proliferative and pro-apoptotic mechanisms [12] and display anti-invasive and anti-migratory effects through reducing protease production by tumour cells, immune cells, and endothelial cells [13].

In 2020, the International Agency for Research on Cancer (IARC) monographs classified opium consumption as *carcinogenic to humans* (Group 1) with *sufficient* evidence for carcinogenicity in the urinary bladder, lung and larynx and *limited* evidence for carcinogenicity in the oesophagus, stomach, pancreas, and pharynx [14]. A systematic review and meta-analysis on the carcinogenicity of opium consumption supported a positive association between opium consumption and cancers of the urinary bladder, larynx, lung, oesophagus, pancreas, and stomach [15]. This raised concerns regarding the potential carcinogenicity of pharmaceutical opioids, whether derived from opium or synthesised to mimic the effect and chemical structure of its alkaloids [16]. Importantly, a recent prospective cohort study reported regular use of pharmaceutical opioids at baseline was associated with increased risk for developing cancer in the organs where cancer risk is increased with opium use (i.e. lung, pancreas, bladder, oesophagus, and larynx), but not in other organs (i.e. prostate, breast, colon, ovary, kidney, and brain) [17].

Discrepancies in the results of in vitro studies investigating the modulation of cancer cells by opioids may be attributed to differences in the opioids tested, the opioid concentrations used, and the cell types examined [2]. For instance, morphine was demonstrated to enhance the proliferation of human glioblastoma T98G cells across concentrations ranging from 50 nM to 40 μM [18], whereas it suppressed the proliferation of MCF-7 breast cancer cells at concentrations below 10 μM [19]. Biphalin; a potent opioid agonist, exhibited an inhibitory effect on human glioblastoma T98G cell proliferation, however, morphine displayed the opposite effect and triggered stimulation of cell proliferation in vitro [18]. Methionine enkephalin (Met-Enk), an endogenous opioid peptide, was demonstrated to have dual effects on tumour growth in vitro. Met-Enk exhibited an inhibitory effect on the growth of SK-N-MC human neuroblastoma cell line, conversely, it stimulated the growth of the U-373MG human astrocytoma cell line [20]. Loperamide, an opioid agonist, suppressed the proliferation of human urinary bladder cancer T24 cells [21] while naltrexone, an opioid antagonist, exhibited the opposite effect by enhancing the proliferation of T24 cells [22]. Additionally, 6-hour exposure to morphine in HCT116 human colon cancer cell line enhanced proliferation, however, this proliferative effect was no longer observed in cells exposed to morphine for 24 hours [23]. While variations in the concentrations employed, durations of exposure, and the specific opioid molecules and receptors involved have been suggested as reasons underlying the discrepancies in the literature, the organ specificity of the effects of opioids on in vitro tumour progression remains unexplored.

This systematic review aimed to examine the role of opioid exposure on tumour growth and metastasis in vitro to determine if opioids differentially modulate cultured cancer cell behaviour depending on the organ of origin of the tumour cells. By addressing this notable gap in the literature, this review offers insights into the modulation of cancer progression by opioids, with significant implications for oncology and pain management.

## Methods

### Literature search and inclusion criteria

We conducted a systematic review of the published literature to identify research articles that evaluate the effect of opioids on tumour growth and metastasis in vitro. This systematic review (registered in the Open Science Framework: osf.io/xyg9p) followed the Preferred Reporting Items for Systematic Reviews and Meta-Analyses (PRISMA) guidelines. An electronic search was conducted by searching three bibliographic databases: PubMed, Embase, and Web of Science. The search included full-text, peer-reviewed English research articles from the inception of the database through 28 February 2024. The search strategy was developed according to PICO (Population: cancer cell culture; Intervention: opioids, opiates; Comparator: control, no opioids; Outcome: growth, proliferation, viability, apoptosis, cytotoxicity, metastasis, migration, invasion). All clinical studies, review articles, conference abstracts, and case reports were excluded, while research articles were only excluded if they involved the use of opium.

Opioids (N02A), as classified under the WHO Anatomical Therapeutic Chemical (ATC) classification system, comprise strong analgesics of the opiate type and analgesics with similar structure or action [24]. However, certain opioids are classified differently based on their therapeutic use in cough suppressants (R05DA), such as codeine, or antipropulsives (A07DA), such as loperamide [24]. The search included the names of the opioids and opiates listed in WHO ATC codes Index (Supplementary data Table 1), as well as the terms opioid* or opiate* AND cancer* OR tumor* OR tumour* OR carcino* OR malignan* OR neoplas* AND growth OR proliferat* OR viability OR apoptosis OR survival OR metastasis OR migration OR angiogenesis OR invasion OR cytotoxicity AND in vitro OR ex vivo OR cell culture* OR tissue culture* OR cancer cell. The following MeSH terms were used in PUBMED: (“Analgesics, Opioid” OR “Opiate Alkaloids” OR “Morphine” AND “Neoplasms” AND “Apoptosis” OR “Neoplasm Metastasis” OR “Neovascularization, Pathologic” OR “Neoplasm Invasiveness” AND “In Vitro Techniques” OR “Cell Culture Techniques” OR “Cells, Cultured”), and in EMBASE, Emtree terms included (“narcotic analgesic agent” AND “neoplasm” AND “oncogenesis and malignant transformation” AND “in vitro study”). The PubMed search included ‘all fields’ while the Web of Science and Embase searches included ‘title/abstract/keywords.’ The Embase search excluded conference abstracts and review articles to eliminate irrelevant studies and refine the search.

### Data extraction

The information extracted included: (a) cancer cell lines (species of origin [e.g. human], organ of origin [e.g. liver], tumour type [e.g. hepatocellular carcinoma]; (b) opioid exposure (opioid name [e.g. morphine], opioid type [e.g. agonist], concentration [e.g. nM], duration of exposure [e.g. 24 hours]); (c) outcome measures (Assay type [e.g. MTT], Assay details [e.g. proliferation]); (d) quantification of assay [e.g. decreased proliferation]); (e) Statistical test present and statistical significance (yes or no).

This information was then used to categorise organs, based on the IARC monograph volume 126 [14]. Organs where opium has been associated with sufficient (lung, bladder, larynx) or limited (pancreas, pharynx, stomach, oesophagus) evidence for carcinogenicity were categorised as ‘Associated with opium use (AWOU)’. Organs with no evidence for carcinogenicity (colon) or not evaluated (adrenal gland, ascites, blood, bone, brain, breast, cervix, connective tissue, kidney, liver, muscle, oral, ovary, pelvis, pituitary gland, prostate, skin, spleen, thyroid, uterus, endometrium) were categorised as ‘Not associated with opium use (NAWOU)’. Contaminated and misclassified cancer cell lines (i.e. CNE-2, SUNE1) that are described as hybrids including a cell line of unknown origin, or SK-HEP-1, which is now reported to have arisen from endothelial cells [25] were recorded as N/A and excluded from the data analysis. The outcome measures were simplified to categorise growth, viability, proliferation, apoptosis, and cytotoxicity as ‘growth’ (e.g. a decrease in apoptosis was recorded as an increase in growth); metastasis, migration, and invasion as ‘metastasis’; and clonogenicity and stem cell activity as ‘clonogenicity’. The quantification of assay was simplified into an overall effect category. This included ‘anti-cancer’, where all outcome measures tested (e.g. growth, migration, etc) were associated with a decrease in cancer aggressive features (e.g. decreased growth and decreased migration) or where the decrease in cancer was the only significant outcome (e.g. significantly decreased migration and non-significantly increased proliferation); ‘pro-cancer’, where all outcome measures tested were associated with an increase in cancer aggressive features or where the increase in cancer was the only significant outcome; ‘complex’ where outcome measures were associated with different effects on cancer (e.g. decreased growth and increased invasion), or where there was a biphasic response observed within the same unit category (e.g. increased at 1 μM and decreased at 100 μM), or across different unit categories (e.g. increased at 10 nM and decreased at 1 μM); and ‘no effect’, where outcome measures showed no change in cancer aggressive features (e.g. no change in viability).

Opioids were classified into four categories: opioid small molecule agonists, referred to as ‘agonists’; opioid small molecule antagonists, referred to as ‘antagonists’; opioid peptide agonists, referred to as ‘peptide agonists’; and opioid peptide antagonists. Opioid agonists and peptide agonists were analysed separately due to their differing dose requirements and distinct mechanism of interaction with their associated receptors on tumour cells. The opioid concentrations were categorised as ‘pM’, ‘nM’, ‘μM’, and ‘mM’. Studies where a range of concentrations were tested (e.g. nM to mM) were recorded as the unit(s) causing the overall effect. The duration of exposure for growth and metastasis was categorised as ‘short’ for ≤ three days; ‘moderate’ for three to seven days; and ‘long’ for ≥ seven days. In research articles that tested multiple opioids, cancer cell lines, concentrations, or exposure durations, the data were analysed as individual experiments, where each experiment included one opioid, one cancer cell line, one concentration category, and one duration category.

The studies identified through the initial literature search were combined with those from a separate search focused on animal model studies. Due to the substantial overlap in results from both searches, the studies were screened simultaneously using Covidence software. Title and abstract screening and full text screening were independently conducted by two reviewers, while data extraction was performed by a single reviewer for each model.

### Statistical analysis

The statistical analysis sought to examine the relationships between key variables of opioid exposure and overall cancer effect (‘anti-cancer’, ‘pro-cancer’, ‘no effect’ or ‘complex’) reported in experimental studies. Key exposure variables included: opioid category (‘agonist’, ‘antagonist’, ‘peptide agonist’, ‘peptide antagonist’); whether or not the organ was linked to the carcinogenicity of opium (‘AWOU’, ‘NAWOU’); opioid concentration (‘pM’, ‘nM’, ‘μM’, or ‘mM’) ; and duration of opioid exposure (‘short’ ‘moderate’ or ‘long’). Contingency tables were created comparing variables against the overall cancer effect. Two-sided Fisher’s Exact tests were used to assess the relationship between each exposure variable and overall cancer effect. A multivariable logistic regression model was used to adjust simulateously for all key exposure variables. For this purposes, overall cancer effect was coded as a binary variable with ‘pro-cancer effect’ assigned a value of 1 and ‘no pro-cancer effect’ (i.e. ‘No effect’, ‘Complex’ or ‘Anti-cancer’) assigned a value of 0. Statistical analysis was performed in R-4.4.0 software and pie charts were generated using GraphPad Prism V10.

### Risk of bias assessment and Sensitivity analysis

In the absence of an existing tool to evaluate the risk of bias in cell culture studies, we chose to focus on two quality criteria, namely the reasonableness of concentrations applied on the cells, and the presence of a statistical test to analyse the results. We independently performed the Two-sided Fisher’s Exact tests on the data set excluding the studies that do not report a statistical test, as well as on the data set excluding studies that employed millimolar concentration of opioid agonists and compared the results to those obtained using the complete dataset on the relationship between the organ of origin and the overall cancer outcome. For each opioid category, the results obtained were similar.

## Results

### Study identification

**Fig. 1:**
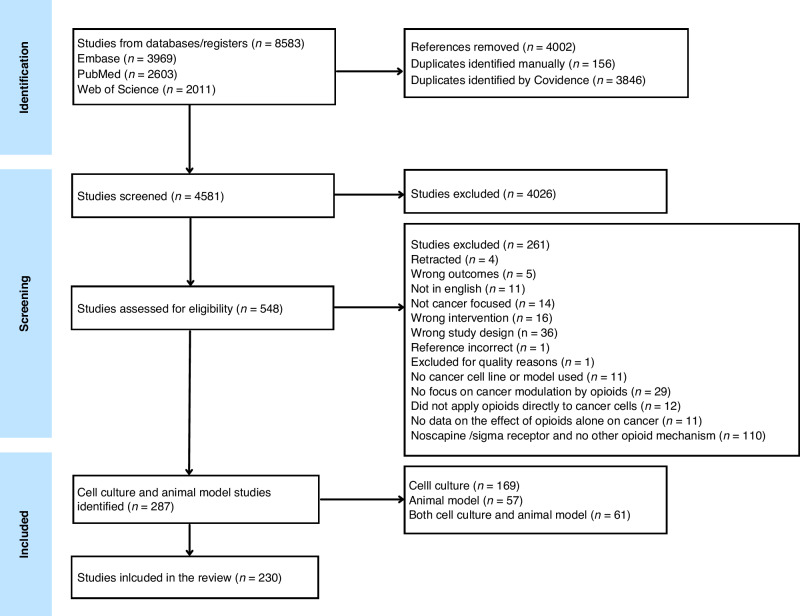
Flowchart of the inclusion and exclusion criteria details.

Figure 1 presents the inclusion and exclusion criteria details. A total of 4581 publications were identified by the literature research after removing duplicates. A total of 548 publications were retained following the initial title and abstract screening. Following the full text review screen, 289 publications were included, which were categorised as ‘cell culture’ or ‘animal model’ or ‘cell culture and animal model’ and underwent data extraction. This systematic review included 230 publications that examined cell culture studies. Studies deemed to be of insufficient quality during the review process were excluded from the analysis. A study, titled ‘The effects of tramadol on cancer stem cells and metabolic changes in colon carcinoma cell lines’ [26] was excluded during the extraction stage. The use of tramadol concentrations in mg/mL for the MTT assay raised concerns regarding the validity of its experimental methods.

### Characteristics of studies

This systematic review included a total of 230 publications, comprising 1465 experiments, which evaluated the effects of opioid exposure on various cancer cell lines. We asked whether the literature supports a dependence of cancer outcomes on the organ of origin of cancer cell lines, opioid concentration, and duration of exposure in cell culture studies. Supplementary data Table 2 and 3 display the publications included in the systematic review and the overall extraction results. All studies were published between the years 1981 and 2024. A total of 347 experiments involved the use of organs classified as ‘AWOU’. Of these, a total of 192 experiments examined the association between opioid exposure and lung cancer, 91 for pancreatic cancer, 23 for bladder cancer, 22 for stomach cancer, 11 for pharyngeal cancer, 7 for oesophageal cancer, and 1 for laryngeal cancer. In contrast, 1114 experiments involved the use of organs classified as ‘NAWOU’, including 198 studies for breast cancer, 100 studies for colon cancer, and 42 studies for liver cancer. The experiments varied in their use of agonists (727), antagonists (212), peptide agonists (517), and peptide antagonists (9).

### Effect of opioid category

The distribution of experimental outcomes (anti-cancer, pro-cancer, no change, complex) based on the opioid category (agonist, antagonist, peptide agonist, peptide antagonist) in cell culture studies is outlined in Table 1. The data indicates that the anti-cancer outcome was the most prevalent across all opioid categories (57%). Agonists and peptide agonists predominantly exhibited anti-cancer activity. Fisher’s test revealed a statistically significant relationship between opioid category and observed cancer outcomes (p = 0.0005). Therefore, subsequent analysis was conducted separately for agonists, antagonists, and peptide agonists using three-way contingency tables (Supplementary data table 4). As only 9 experiments employed peptide antagonists, they were excluded from further analysis. Overall, the analysis showed that while opioids predominantly exhibited anti-cancer outcomes, their specific impact on cancer outcomes was influenced by the category they belong to. The distribution of outcomes for each organ is detailed in Supplementary data table 5.

**Table 1:** The distribution of experimental outcomes based on the opioids category in cancer cell culture studies.

	Anti-cancer	Pro-cancer	No change	Complex	Overall
**Agonist**	472 (65%)	83 (11%)	160 (22%)	12 (2%)	727
**Antagonist**	85 (40%)	29 (14%)	97 (46%)	1 (0%)	212
**Peptide agonist**	276 (53%)	47 (9%)	185 (36%)	9 (2%)	517
**Peptide antagonist**	0 (0%)	1 (11%)	8 (89%)	0 (0%)	9
**Overall**	833 (57%)	160 (11%)	450 (31%)	22 (2%)	1465

### Effect of organ of origin

**Fig. 2:**
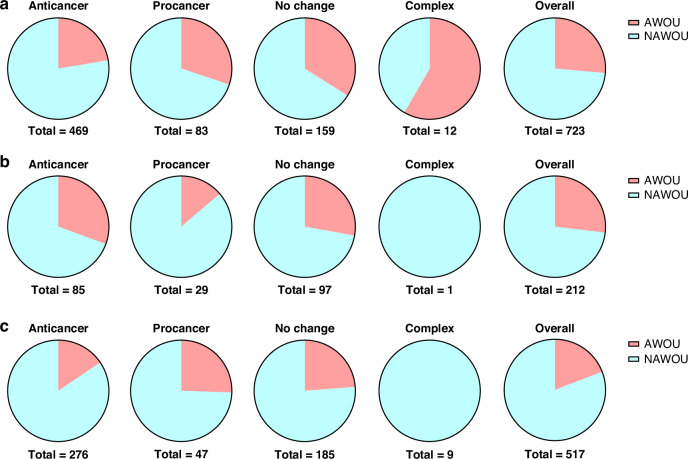
Effect of the organ of origin of cancer cell lines. Distribution of experiments outlining the anti-cancer, pro-cancer, no change or complex effect and the overall distribution based on the organ of origin of cancer cell lines for (**a**) agonists; (**b**) antagonists; and (**c**) peptide agonists in cell culture studies. NAWOU, not associated with opium use; AWOU, associated with opium use.

We investigated whether the organ of origin of cancer cell lines influenced cancer outcomes in cell culture experiments. The distribution of experiments reporting anti-cancer, pro-cancer, no change or complex effects, categorised based on the organ of origin as ‘associated with opium use (AWOU)’ or ‘not associated with opium use (NAWOU)’, is illustrated in Fig. 2. For agonists (Fig. 2a) and peptide agonists (Fig. 2c), but not for antagonists (Fig. 2b), the proportion of experiments using cell lines from ‘AWOU’ organs seemed higher in studies showing pro-cancer or no change effects than in studies showing anti-cancer effects. Specifically, among experiments investigating agonists, the proportion of experiments using cell lines from ‘AWOU’ organs was 30% in studies showing pro-cancer effects but 22% in studies showing anti-cancer effects. Similarly, among experiments investigating peptide agonists, the proportion of experiments using cell lines from ‘AWOU’ organs was 26% in studies showing pro-cancer effects but 16% in studies showing anti-cancer effects.

Fisher’s exact test confirmed a significant dependence between the organ category and cancer outcome for agonists (p = 0.002) and peptide agonists (p = 0.042). However, this was not true for antagonists (p = 0.315). This suggests that the organ of origin of cancer cell lines influences the effect on cancer outcomes for opioid agonists and peptide agonists.

### Effect of opioid exposure duration

**Fig. 3:**
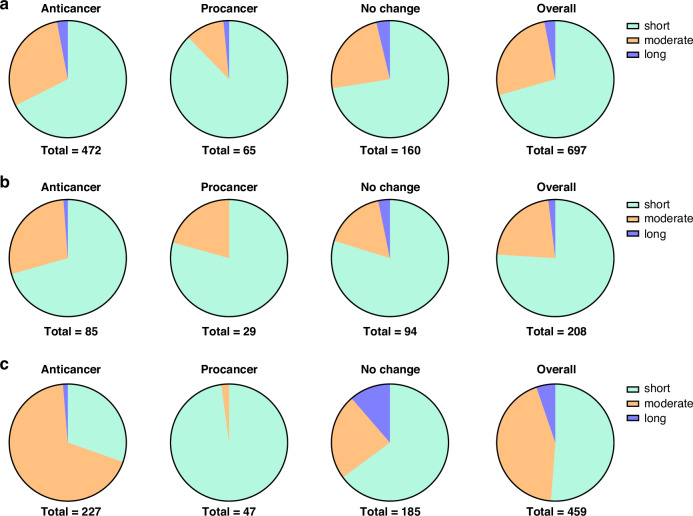
Effect of duration of exposure to opioids. Distribution of experiments outlining the anti-cancer, pro-cancer, no change or complex effect and the overall distribution based on the duration of exposure of cancer cell lines to opioids for (**a**) agonists; (**b**) antagonists; and (**c**) peptide agonists in cell culture studies.

We investigated the influence of the duration of opioid exposure on cancer outcomes in cancer cell culture studies. Figure 3 outlines distribution of experiments reporting anti-cancer, pro-cancer, no change or complex effects based on opioid’s duration of exposure, categorised as ‘short’, ‘moderate’, or ‘long’. Across all categories, the proportion of experiments using short durations was higher in the pro-cancer outcome experiments than in the anti-cancer outcome experiments (e.g., for the agonists, short duration represented 67% of experiments showing an anticancer effect but 88% of studies shoing a procancer effect). Conversely, a long duration of opioid exposure was predominantely associated with anti-cancer or no change outcomes. Fisher’s exact test revealed significant association between the duration of exposure and cancer outcomes for agonists (p = 0.0035) and peptide agonists (p = 0.0005), but not opioid antagonists (p = 0.471). This suggests that the duration of exposure of cancer cell lines to opioid agonists and peptides influences cancer outcomes.

### Effect of opioid concentration

**Fig. 4:**
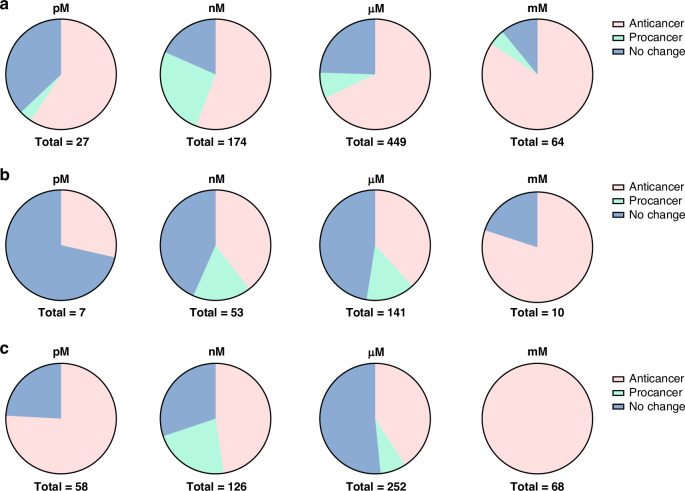
Effect of opioid concentration. Distribution of the experiments outlining the effect of the opioid concentration (pM, nM, μM, mM) on the cancer outcome (anti-cancer, pro-cancer, no change, complex) for (**a**) agonists; (**b**) antagonists; and (**c**) peptide agonists in cell culture studies.

We investigated the influence of the opioid concentrations used in cancer cell experiments on the observed cancer outcomes. Figure 4 outlines the the distribution of experiments employing picomolar (pM), nanomolar (nM), micromolar (μM), or millimolar (mM) concentrations and their cancer outcomes. Overall, it was observed that the proportion of experiments reporting anti-cancer outcomes increased with the concentration of opioids. Fisher’s exact test revealed a significant association between the opioid concentrations employed and cancer outcomes for agonists (p = 0.0005) and peptide agonists (p = 0.0005), but not for antagonists (p = 0.334). Overall, there is evidence to suggest that the concentrations of opioid agonists used in cancer cell culture experiments influence cancer outcomes. Table 2 summarises the Fisher’s exact test values obtained when analysing each contingency table.

**Table 2:** Overview of p-values from Fisher’s exact test.

Contingency Table	*p* value
Opioid Category v Cancer Outcome	0.0005
Concentration v Cancer Outcome, “agonist”	0.0005
Concentration v Cancer Outcome, “antagonist”	0.334
Concentration v Cancer Outcome, “peptide agonist”	0.0005
AWOU v Cancer Outcome, “agonist”	0.002
AWOU v Cancer Outcome, “antagonist”	0.315
AWOU v Cancer Outcome, “peptide agonist”	0.042
Duration v Cancer Outcome, “agonist”	0.0035
Duration v Cancer Outcome, “antagonist”	0.471
Duration v Cancer Outcome, “peptide agonist”	0.0005

### Logistic regression

Multivariable logistic regression analysis was conducted for agonists and peptide agonists combined, or agonists alone, with the latter providing the best fit model (AIC = 407) (Supplementary table 6). The data reported below are from the logistic regression for the agonists alone (reference levels, dose: ‘millimolar’; duration: ‘moderate’). After simultaneous adjustment for all assessed key exposure variables, the variables significantly and positively influencing the probability of the experiment outcome being classified as ‘pro-cancer’ were a short duration of opioid exposure (OR 5.4, 95% CI 2.3 – 12.7, p = 9 ×10^−5^), nanomolar opioid concentrations (OR 9.0, 95% CI 2.0 – 39.6, p = 0.00372) and organs categorised as associated with opium exposure ‘AWOU’ (OR 1.9, 95% CI 1.1 – 3.3, p = 0.02883).

## Discussion

The notion that opioids promote cancer progression has been a long-standing concern, especially given their critical role in managing chronic non-cancer and cancer-related pain in clinical settings. Studies discussing the use of opioids during surgery and management of cancer pain frequently cite literature suggesting an association between opioid administration and cancer recurrence. However, in agreement with recent evidence taking into account cancer type and individual tumour characteristics [27], our findings show that the inhibitory effects of opioids on cancer-related features, such as viability, proliferation, migration, and invasion, are most prevalent in experimental cell culture studies, representing 57% of the reviewed literature, with only 11% reporting pro-cancer effects for opioids. This review further shows that in cell culture studies, pro-cancer outcomes were more commonly observed with short durations of opioid exposure or low opioid concentrations. Importantly, our review provides new evidence indicating that the contrasting findings on pro-cancer vs. anti-cancer effects of opioids may partly depend on the organ of origin of the assessed cancer cells; experimental studies involving cancer cells derived from organs identified by IARC monographs as having increased cancer risk with opium use [14] were more likely to report pro-cancer outcomes in relation to opioid exposure.

This systematic review provides evidence that opioid category (agonist, antagonist, peptide agonists) is associated with cancer outcomes (contingency table), which was unsurprising since agonists and antagonists are not expected to lead to the same outcome. Antagonists are unlikely to exert much of an effect in vitro unless the receptor exhibits constitutive activity or an endogenous agonist is present. In the logistic regression for agonists, organs where cancer risk is associated with opium use ‘AWOU’, nanomolar opioid concentrations and short exposure durations were significantly and positively associated with reporting pro-cancer effects (*vs* no pro-cancer effects). This suggests that in addition to the known carcinogens present in opium, such as polycyclic aromatic hydrocarbons [14], opioid alkaloids may partly contribute to the increased cancer risk associated with opium use. The organ-specific modulation of cancer by opioids may be attributed to several potential mechanisms. This includes the differential expression of opioid receptors; mu (MOR), kappa (KOR), and delta (DOR), by epithelial and cancer cells in different organs [28], which could in theory modulate cell response to opioid exposure, influencing cancer-related features [29]. For instance, MOR is overexpressed in human non-small cell lung cancer (NSCLC) cells, where morphine, a MOR agonist, increased in vitro Lewis lung carcinoma (LLC) cell growth, while methylnaltrexone, a MOR antagonist, attenuated LLC growth [29]. In triple negative breast cancer (TNBC) tumour samples, KOR and DOR expression was increased compared to normal breast tissue [30]. However, in most cancer cell lines in vitro [31] and in cancer cells in vivo [30] the expression of opioid receptors, and more specifically the MOR, is exceedingly low or nonexistent, making receptor expression unlikely to mediate the organ-specificity of the effect of opioids on cancer. Even when analysing tumour tissue (which includes cancer and non-cancer cells of the TME) solid tumour OR expression is extremely low, and in any case not associated with long term oncologic outsomes [32]. Rather than mere OR expression, recent clinical evidence focusing on tumour OMICS points to oncogenic pathways as a mechanism explaining differential effects of opioids in distinct cancer types [33–35].

Although our review only assesses data on the effect of opioids on cancer cells, it is important to note that rather than a direct action on the cancer cells, organ-specific opioid interactions likely involve mechanisms within the tumour microenvironment (TME), which comprises stromal cells, fibroblasts, endothelial cells, and immune cells [36]. This would not have been captured by our systematic review as we focused on cancer cells. The TME, which varies across different organs, plays a key role in cancer progression, particularly immune-escaping, and distant metastasis [37, 38]. The tumour-killing immune microenvironment includes cytotoxic T lymphocytes (CTLs), natural killer (NK) cells, helper T (Th) cells, and M1-type tumour-associated macrophages (TAMs), while the immune suppressive microenvironment comprises M2-type TAMs and myeloid-derived suppressor cells (MDSCs) [36, 37]. Opioids may convert the immune-suppressive TME to an immune-infiltrating environment, thereby enhancing the anti-tumour immune response [37]. For instance, in lung carcinoma cells, MET-ENK treatment increased the infiltration of CTLs, Th cells, NK cells, and M1-type TAMs, while reducing the infiltration of MDSCs and M2-type TAMs into the TME, contributing to anti-tumour effects [37]. Emerging clinical evidence using genomic biomarkers indicates that immunomodulation indeed underlies tumour type specificity of the effects of opioids [39]. Opioids have also been shown to modulate angiogenesis, a critical process in cancer progression, acting as pro- or anti-angiogenic factors depending on their concentration and receptor affinity [8]. The underlying processes include the direct stimulation of endothelial cells, immune cells, tumour cells, keratinocytes and fibroblasts, and promoting the release of angiogenic factors such as nitric oxide (NO), prostaglandin E2 (PGE2), and vascular endothelial growth factor (VEGF) [8].

Our results show that short durations of opioid exposure were associated with more frequently reporting pro-cancer outcomes in cell culture studies. The duration of opioid exposure in cell culture studies may modulate opioid receptor tolerance and desensitisation, with prolonged exposure leading to tolerance and reducing opioid efficacy [40]. The molecular mechanisms underlying opioid receptor desensitisation involve phosphorylation, receptor uncoupling, internalisation, and post-endocytic fate of the receptor [40]. Multiple factors, such as the cellular model, agonist concentration, duration of exposure, opioid receptor expression and signalling pathways involved, influence receptor desensitisation [40]. Opioid receptors can simultaneously activate multiple signalling pathways, such as adenylyl cyclase (ACs), mitogen-activated protein (MAP) kinases, or ion channels, which modulates the level of desensitisation [40]. While the duration of opioid exposure may modulate cancer outcomes, it is difficult to correlate in vitro exposure durations to clinical settings, where opioid administration can be prolonged and is influenced by pharmacokinetic and pharmacodynamic processes.

We further observed a biphasic trend in the literature, whereby studies employing low opioid concentrations (in the nanomolar range), more frequently reported pro-cancer outcomes when compared to studies using higher opioid concentrations (in the millimolar range). This may be explained by the cytotoxicity of higher opioid concentrations [41]. At higher concentrations, opioids have been shown to inhibit tumour necrosis factor-alpha (TNF-α) expression [42, 43] and stimulate the production and release of nitric oxide (NO) and reactive oxygen species (ROS), both of which exert direct and indirect tumoricidal effects [44]. This biphasic concentration effect seen in the literature is consistent with individual studies that show a biphasic effect within the same experiment [41, 45–47]. Notably, dezocine, a mixed MOR and KOR agonist-antagonist, with a higher affinity for MOR, showed the opposite trend where it inhibited tumour growth at low concentrations but stimulated growth at higher concentrations [48, 49]. The mechanisms underlying the biphasic concentration effect may include differential receptor subtype activation [50], receptor desensitisation and internalisation at higher concentrations, as well as alterations in intracellular signalling pathways [40, 51]. Additional biochemical reasons include the shift from antagonist to agonist activity due to the activation of lower-affinity receptors, increased cellular clearance at higher concentrations, and receptor downregulation due to saturation and loss of receptor specificity at higher concentrations [52]. For instance, when opioid concentrations surpass the saturation of their primary targets, they may engage other lower-affinity receptors such as Toll-like receptor 4 (TLR4), which is known to control aggressive features in cancer cells [53].

To contextualise the findings from this review in a clinical setting, we compared the opioid concentrations used in vitro to those typically detected in clinical practice. Clinical studies report circulating opioid concentrations ranging between 1 nanomolar to 1 micromolar for commonly used opioids such as buprenorphine, fentanyl, methadone, morphine, oxycodone, and tramadol [54]. These concentrations predominately fall within the nanomolar range, which is where the literature indicates opioids are most commonly associated with cancer progression in cell culture studies. Interestingly, exclusion of studies that used millimolar (mM) concentrations in our analyses had no impact on the overall findings (data not shown).

This is the first systematic review investigating the organ-specific modulation of cancer by opioids in cell culture studies. It is important to note that the in vitro direct effects of opioids only partially contribute to the in vivo effects of these drugs. By evaluating whether the cell culture literature supports existing epidemiological data, we provide a comprehensive reference for future research, offering insights that can inform future experimental approaches and influence clinical practices in cancer care. Our review highlights heterogeneity among the included studies, which is evident is several aspects. There is a notable imbalance in the cancer types studied, for instance, lung cancer was more extensively studied compared to other organs where cancer is associated with opium use. This may have introduced bias, as the outcomes from heavily studied cancer types may not generalise across other organ systems. Variability in the number of opioids studied within each category (agonists, antagonists, and peptides), as well as differences in opioid concentrations and durations of exposure, further contributes to the observed heterogeneity. This heterogeneity precluded the possibility of conducting a meta-analysis. Moreover, categorisation decisions, such as those based on opioid concentrations and exposure durations, though necessary for consistency when comparing results across studies, may have introduced bias.

Given the widespread use of opioids in cancer care, understanding their impact on tumour progression is essential for optimising treatment strategies. The significance of this research lies in its potential to advance our understanding of the complex relationship between opioids and cancer, with significant implications for clinical use, research strategies, and therapeutic advancements. The outcomes of this systematic review have the potential to guide future research, ultimately improving patient outcomes and quality of life in cancer care.

This systematic review supports the findings of the IARC monographs, volume 126, which classified opium consumption as *carcinogenic to humans* (Group 1) with *sufficient* evidence for carcinogenicity in the urinary bladder, lung and larynx and *limited* evidence for carcinogenicity in the oesophagus, stomach, pancreas, and pharynx. Our results confirm a positive association between opioid exposure and in vitro growth or metastasis of cancer cells isolated from organs where cancer risk is associated with opium use, namely the lung, bladder, larynx, pancreas, pharynx, stomach, and oesophagus. We further observed an influence of the concentration or duration selected by the experimentalists in determining in vitro cancer aggressive features.

## Supplementary information

Supplementary material

## Data Availability

The datasets analysed in this review are available as supplementary material.
